# Cutaneous tuberculosis and HIV infection at a referral centre in Rio de Janeiro, Brazil

**DOI:** 10.1590/0074-02760180184

**Published:** 2018-07-26

**Authors:** Danielle Mann, Flávia Marinho Sant’Anna, Carolina Arana Stanis Schmaltz, Dayvison Francis Saraiva Freitas, Valeria Cavalcanti Rolla, Solange Cesar Cavalcante, Maria Clara Gutierrez-Galhardo

**Affiliations:** 1 Fundação Oswaldo Cruz-Fiocruz, Instituto Nacional de Infectologia Evandro Chagas, Laboratório de Pesquisa Clínica em Dermatologia Infecciosa, Rio de Janeiro, RJ, Brasil; 2 Fundação Oswaldo Cruz-Fiocruz, Instituto Nacional de Infectologia Evandro Chagas, Laboratório de Pesquisa Clínica em Micobacterioses, Rio de Janeiro, RJ, Brasil

**Keywords:** cutaneous tuberculosis, Mycobacterium tuberculosis, HIV, AIDS, immune reconstitution inflammatory syndrome.

## Abstract

**BACKGROUND:**

Cutaneous tuberculosis (CTB) is a rare extrapulmonary form of tuberculosis (TB). Despite the increase in the number of cases of TB and HIV, few cases of CTB have been reported.

**OBJECTIVE:**

To describe CTB cases among patients with HIV infection from a cohort with tuberculosis.

**METHODS:**

We describe a series of 15 CTB and HIV cases, based on secondary data from 2000 to 2016. Diagnosis was based on isolation of *Mycobacterium tuberculosis* in culture or clinical response to anti-tuberculous treatment associated with positive smear or histopathologic findings from affected skin or an adjacent lymph node.

**FINDINGS:**

Scrofuloderma was present in 12 (80%) patients and solitary gumma in three (20%) patients. One case of scrofuloderma was associated with papulonecrotic tuberculid. Seven (46.6%) patients had pulmonary TB. Diagnosis was based on culture in nine patients (60%). The median CD4 cell count was 262 cells/µL. All patients were cured at the end of treatment (median time 6 months). Three patients presented with immune reconstitution inflammatory syndrome.

**CONCLUSIONS:**

In this study, CTB associated with HIV infection presented as localised forms or in association with pulmonary TB. In patients with HIV who have subacute and chronic skin lesions, CTB should be considered in differential diagnosis, which may represent a good opportunity for early diagnosis of active TB.

The association between tuberculosis (TB) and the human immunodeficiency virus (HIV) remains an important issue for public health worldwide. Individuals with HIV infection are 26 times more likely to have active TB than those without HIV.^1^ ^,^ ^2^

According to the World Health Organization (WHO) Global TB Report for 2017, approximately 10.4 million individuals became ill with TB in 2016.^1^ Brazil ranks at the 20th position according to the classification of disease burden and the 19th in terms of TB/HIV coinfection rates among countries that represent 87% of the world’s cases of TB, with an incidence rate of TB and TB/HIV of 42/100,000 and 5.2/100,000 inhabitants, respectively, and a general mortality rate of 2.6/100,000 for TB and 0.9/ 100,000 for HIV/TB.^1^ ^,^ ^3^

Cutaneous tuberculosis (CTB) is a rare extrapulmonary form of TB and remains one of the least studied. It is estimated that 14% of TB patients present the extrapulmonary form and, among these, 1-2% have cutaneous presentation.^4^ Skin involvement may result from exogenous inoculation, spread from an adjacent focus, or it may be secondary to lymphohaematogenous dissemination.^5^ ^,^ ^6^ The clinical presentations are pleomorphic and are classified as tuberculous chancre, warty TB, lupus vulgaris, tuberculous gumma, acute miliary TB, orificial TB, and scrofuloderma. There are also the tuberculids, considered immunologic reactions to *Mycobacterium tuberculosis*, which are classified as papulonecrotic tuberculid, lichen scrofulosorum, and erythema induratum of Bazin.^5^ ^-^ ^7^

Despite the increasing number of TB cases associated with HIV infection, few cases of CTB have been reported. We found 87 cases of CTB and HIV described in 12 papers in the literature since the beginning of the HIV epidemic, up to and including November 2017.^6^ ^,^ ^8^ ^-^ ^18^ This study describes the cases of CTB among patients with HIV infection who were patients followed at a referral centre for TB and HIV and by a team of dermatologists (in partnership) in the city of Rio de Janeiro, Brazil.

MATERIALS AND METHODS

This study was approved by the Ethics Committee of Evandro Chagas National Institute of Infectious Diseases (INI)/Oswaldo Cruz Foundation (Fiocruz) (CAAE: 57208016.9.0000.5262). An informed consent form was obtained from all patients who had entered the cohort study since 2016. This study is based on secondary data; all cases of cutaneous TB were reviewed by the group of the TB laboratory and dermatology. Before this review, a statement of responsibility was signed by the principal investigator ensuring the confidentiality of the data.

We describe a series of 15 CTB and HIV cases based on secondary data of a prospective ongoing cohort study, ongoing since 2000, conducted at INI/Fiocruz in Rio de Janeiro, Brazil. During this period, 601 cases of TB and HIV were attended to. We reviewed the electronic medical records (EMR) of all cases classified as CTB during the period from 2000 to 2016.

The inclusion criteria were: age ≥ 18 years with (a) *M. tuberculosis* isolated on culture of the affected skin or a contiguous focus (by biopsy or aspiration); or (b) clinical response to anti-tuberculous treatment associated with positive smear or histopathology showing chronic granulomatous infiltrate with necrosis, from clinical specimens of the affected skin or a contiguous focus (by biopsy or aspiration). The exclusion criterion was a lack of information in the medical records.

All clinical specimens that underwent TB diagnosis were submitted to a microbiologic examination that included acid-fast bacilli (AFB) detection using the Ziehl-Neelsen technique and culture in Löwenstein-Jensen medium, and histopathological staining with haematoxylin-eosin (H&E) and Wade stains. In the case of biopsy specimens, they were divided into two fragments: one was fixed in 10% buffered formalin, embedded in paraffin, and stained with H&E and Wade for histopathological examination; the other was kept in sterile saline, triturated, and then submitted for microbiologic examination.

All patients were monitored according to a previously defined protocol that included routine laboratory tests of blood cell count; serum levels of creatinine, urea, uric acid, liver enzymes, and albumin; CD4 cell count; HIV viral load; hepatitis B and C serology; chest radiographs; and sputum smear and mycobacteria cultures. A tuberculin skin test (TST) was also performed and was considered positive with induration ≥ 5 mm. The beginning of follow-up was defined as the date of the first prescription of anti-tuberculous therapy, and subsequent follow-up was scheduled 15, 30, 60, 90, 120, and 180 days later. In cases where cure was not achieved after 6 months, additional follow-up was required. Routine laboratory tests were performed at each follow-up visit. CD4 cell count and HIV viral load were measured at baseline and after the introduction of combined antiretroviral therapy (cART), to evaluate virologic control and identify immune reconstitution inflammatory syndrome (IRIS). Sputum smear and mycobacteria cultures were repeated monthly until negative results were obtained.

Patients included in the study were managed following the Brazilian guidelines for TB and HIV treatment.^19^ ^,^ ^20^ The diagnosis of HIV infection was established by serologic detection of specific antibodies using enzyme-linked immunoassay (ELISA) plus confirmation with immunofluorescence or western blot, as recommended by the Brazilian Ministry of Health.^20^ ^,^ ^21^ Paradoxical IRIS was considered a documented worsening of signs or symptoms of CTB during appropriate anti-tuberculous treatment and following the initiation of antiretroviral therapy, not explained by any other disease or by an adverse effect of drug therapy, despite improved immune function. Unmasking IRIS was considered when defined if the patient was not receiving treatment for TB when cART was initiated and then presented developed CTB within 3 months of starting cART, with heightened intensity of clinical manifestations. Patients received a regimen of three drugs for TB (rifampicin, isoniazid, pyrazinamide; RHZ) until 2009 in Brazil; ethambutol was subsequently added in a fixed dose combination (RHZE).^19^

Clinical cure was defined as the absence of active skin lesions. Usually, the standard treatment was for 6 months; however, if there were signs of clinical activity, the treatment was maintained until clinical cure. Sociodemographic, clinical, laboratory, first positive HIV serology, and cART data were collected from the patient’s EMR. The data obtained were stored in Microsoft Excel® version 2016 and R-Project version 3.3.3 was used for descriptive analysis, such as frequencies for categorical variables and summary measures (mean, median and range) for continuous variables.^22^ The Shapiro-Wilk test showed that the variable time to signs of clinical improvement followed normality, considering p-value < 0.05.

RESULTS

In this study, the diagnosis of CTB was based on the identification of *M. tuberculosis* in cultures from nine patients (60%); or based on clinical response to anti-tuberculous treatment and positive smear (26.7%, n = 4) or lymph node histopathologic (13.3%, n = 2) findings in six (40%) patients.

There was a predominance of male patients (9/60%) ranging in age from 24 to 52 years (median age 33 years), and non-white individuals (11/73.3%) (Table I). Ten (66.7%) patients were from the municipality of Rio de Janeiro and five (33.3%) were from other cities in the state. Ten (66.7%) patients had monthly income of up to USD 571 per month; these data were missing for five (33.3%) patients. Twelve (80%) patients had completed at least 9 years of schooling, and two (13.3%) had completed less than 9 years; no education data were available for one patient. At the time of CTB diagnosis, two (13.3%) patients had a history of excessive alcohol consumption and one (6.6%), a history of illicit drug use. One patient (case 6) had a previous history of pulmonary TB and another (case 5) had relatives with TB. No patients had received previous treatment for TB latent infection. All patients, except two (cases 1 and 6), had constitutional symptoms such as weight loss (86.7%), fever (80%), adynamia (66.7%), and sweating (40%).

CTB presented as scrofuloderma in 12 (80%) patients and as solitary tuberculous gumma in three (20%) patients (Table II). Scrofuloderma was associated with lymph node involvement in 11 cases (10 in the cervical region and one in the supraclavicular lymph node). One patient (case 14) also presented papules, pustules, crusts, and atrophic scars on the back, clinically diagnosed as papulonecrotic tuberculid (Fig. 1A-B). In Case 4, scrofuloderma was associated with skeletal TB, evidenced by lytic lesions of the vertebral bodies from T9 to L2 and with paravertebral masses visible by computed tomography. The patient had paresthesia in the right thigh for several months.

Three patients who presented with gumma were female; their lesions were small, isolated, similar to a furuncle and of a transitory nature, and were partially resolved with diagnostic aspiration (Fig. 2). In two patients, the lesions were localised in the subcutaneous tissue of the mammary region, which was classified as mammary TB.

**TABLE I:** Demographic aspects, HIV laboratory parameters and combination antiretroviral therapy (cART) of the patients enrolled in this study

Case	Age	Sex	Race	CD4	Log	cART /time of use^*a*^
1	48	F	NW	441	-	AZT, 3TC, EFZ/2 years
2	33	F	NW	6	5.453	AZT, 3TC, EFZ/4 months
3	52	F	NW	1,846	2.476	TDF^*b*^ ,3TC, EFV/8 years
4	30	M	W	282	-	AZT, 3TC, EFZ/1 year
5	32	M	NW	19	5.032	No
6	51	M	W	571	-	AZT+3TC+EFV/15 years
7	28	F	NW	41	5.610	No
8	33	M	NW	329	4.681	No
9	29	M	NW	369	3.544	No
10	46	M	W	84	4.546	No
11	24	F	NW	432	4.439	No
12	43	M	NW	9	5.084	3TC+EFV+ddI+LPV/r/2 months
13	41	F	NW	195	-	No
14	32	M	NW	98	4.631	No
15	28	M	W	242	3.361	3TC+TDF+EFV/<1 month

ART: antiretroviral; AZT: zidovudine; ddI: didanosine; EFV: efavirenz; F: female; Log: viral load values in a logarithmic scale; LPV/r: lopinavir/ritonavir; M: male; NW: non-white; TDF: tenofovir; W: white; 3TC: lamivudine; *a*: time between the onset of cART and anti-tuberculosis treatment; *b*: use of stavudine for 5 years before TDF.

In seven cases (46.6%), CTB was associated with pulmonary TB (sputum positive culture in six patients and one diagnosed by alterations visible in their chest radiograph that improved with treatment); however, four patients (57.1%) had no respiratory symptoms. Typical chest radiography findings were present in five patients (bilateral diffuse or perihilar and pericardial and/or micronodular, nodular, reticular, and mixed infiltrates); the other two patients had normal radiographic examination findings. TST was not performed in some patients because the test was unavailable in Brazil during some periods. TST was positive in three (case 2: 10 mm; case 3: 49 mm and case 11: 18 mm) of six patients tested (cases 1, 4, and 10 were negative). Eight patients were classified as having disseminated TB.

The median CD4 cell count was 262 cells/µL (ranging from 6 to 1,846 cells/µL) (Table I). Seven patients (46.6%) had a diagnosis of CTB and HIV infection at the same time (cases 5, 7, 9, 10, 11, 14, and 15).

Median time from the onset of CTB symptoms and the initiation of anti-tuberculous therapy was 3 months (ranging from 0.6 to 12 months). Eight patients received the 3-drug regimen (RHZ) and seven patients received the 4-drug regimen (RHZE). Ten patients were treated for 6 months and five patients required a longer treatment time to heal their lesions: case 7 (10 months); case 15 (8 months); and cases 4, 6, and 13 (9 months). Ten patients (66.7%) experienced adverse reactions, which were mainly gastrointestinal. However, only two patients (cases 7 and 12) required temporary suspension of drugs (due to pruritus and hepatotoxicity, respectively); in both these patients, reintroduction of the drugs was successful. All patients were cured at the end of treatment. Nine patients (60%) were receiving cART before the appearance of CTB and three of them (cases 2, 12, and 15) had unmasking IRIS. These three patients had onset of CTB symptoms up to 2 months after the initial use of cART, associated with an increased CD4 cell count and a decreased HIV viral load (of at least 2.9 log_10_) (Table I). Because of substantial inflammatory adenomegaly associated with IRIS, one patient (case 12) required the use of prednisone for 8 months.

DISCUSSION

In this study, most patients with CTB/HIV were male, non-white, and had low education levels, following the profile of individuals living with HIV in Brazil.^20^ The 15 cases of CTB represented 2.5% of TB/HIV cases in the INI attended over a period of 17 years. Different from other opportunistic illnesses, TB can occur in patients with HIV who have widely varying CD4 counts, from those with preserved immunity to different degrees of cellular immunity impairment.

In our study, lymph node scrofuloderma was the predominant clinical form of CTB, as previously described.^12^ ^,^ ^14^ Scrofuloderma corresponds to involvement and rupture of the skin over a contiguous focus of TB; however, it can occur in other sites, such as the glands, tear ducts, and epididymis, among others.^6^ Cervical lymphadenitis is the most frequent form of extrapulmonary TB and signs of worsening, such as node enlargement with pain, suppuration, sinus formation, and appearance of new nodules, can occur in 25-30% of cases, during and after treatment.^23^ Paraspinal cold abscess is the second most common focus of scrofuloderma, and it is a complication of vertebral TB in 50% of cases.^23^ Skeletal system involvement occurs in 1-3% of all TB cases, and nearly 30% of these are in patients with HIV.^24^ Although our case 4 presented neurologic symptoms (paresthesia), the diagnosis of CTB was made prior to that of Pott’s disease.

**TABLE II:** Clinical features and diagnosis of cutaneous tuberculosis of patients with HIV infection attended at Instituto Nacional de Infectologia Evandro Chagas/Fiocruz, Rio de Janeiro, Brazil, from 2000 to 2016

Case	Time of evolution (months)	Material	Microbiological exam (AFB/culture)	Clinical form	Localisation	Other affected site
1	ND	Aspirated abscess	(+)/(-)^**^	Gumma	Left breast	
2	< 1	Aspirated abscess	(+)/(+)	Gumma^*^	Left thigh	
3	12	Aspirated abscess	(+)/NP^**^	Gumma	Right breast	
4	12	Aspirated abscess	NP/(+)	Scrofuloderma	Spine paravertebral abscess	Supraclavicular lymph node
5	3	Aspirated lymph node abscess	(+)/(+)	Scrofuloderma	Cervical lymph node	
6	5	Lymph node fragment	(-)/(-)^***^	Scrofuloderma	Cervical lymph node	
7	3	Aspirated lymph node abscess	(+)/(+)	Scrofuloderma	Cervical lymph node	Lung
8	ND	Aspirated lymph node abscess	(+)/(+)	Scrofuloderma	Cervical, axillary lymph node	Lung
9	2	Aspirated lymph node abscess	(+)/(+)	Scrofuloderma	Cervical lymph node	
10	ND	Lymph node fragment	(+)/NP^**^	Scrofuloderma	Supraclavicular lymph node	Lung
11	4	Lymph node fragment	(-)/(+)	Scrofuloderma	Cervical lymph node	Lung
12	2	Aspirated lymph node abscess	(+)/(+)	Scrofuloderma^*^	Cervical lymph node	Lung
13	4	Aspirated lymph node abscess	(+)/(+)	Scrofuloderma	Cervical lymph node	Lung
14	8	Lymph node fragment	(-)/(-)^***^	Scrofuloderma/ Papulonecrotic tuberculide	Cervical lymph node	
15	ND	Aspirated lymph node abscess	(+)/(-)^**^	Scrofuloderma^*^	cervical lymph node	Lung

AFB: acid-fast bacilli; ND: no data; NP: not performed; *: cutaneous tuberculosis (CTB) associated to immune reconstitution inflammatory syndrome. The diagnosis of CTB was based on clinical response to antituberculous treatment associated to smear** of clinical specimens or lymph node histopathologic findings***.

Tuberculous gumma was described in a few of our patients. Gumma is a rare form of haematogenous TB that arises from the reactivation of latent foci, usually when the host has low resistance or in immunosuppressed patients, resulting in single or multiple lesions.^6^ ^,^ ^7^ Gumma occurs more often in the extremities; however, in two cases presented here, gummas were localised in the mammary region. Breast TB (BTB) is a very rare form of extrapulmonary TB, even in endemic areas of the world. The clinical presentation of BTB is usually associated with pain in the breast, breast lumps, and nipple discharge or abscess, as seen in our cases.^25^ TB gumma shares clinical and histologic features with scrofuloderma but has different mechanisms of infection.

**Fig 1:**
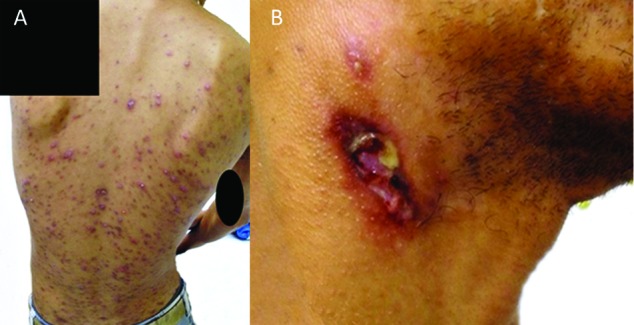
patient with associated forms of cutaneous tuberculosis. (A) Papulonecrotic tuberculid on the back. (B) Scrofuloderma on the cervical region (Case 14).

**Fig 2:**
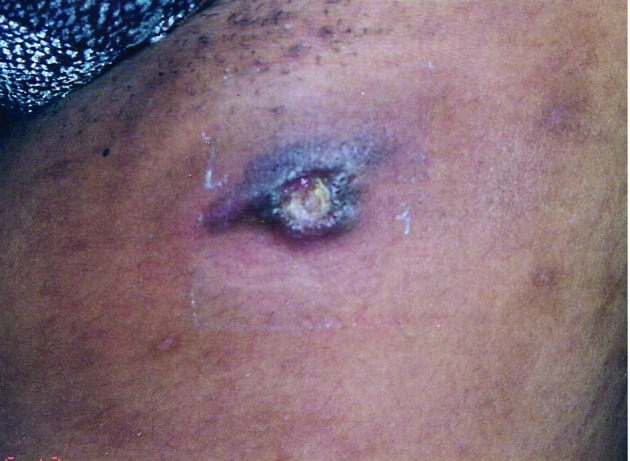
tuberculous gumma on the anterior left thigh (Case 2).

Papulonecrotic tuberculid is associated with other sites of TB in up to 75% of cases and generally represents a good immunological response to *M. tuberculosis*.^6^ ^,^ ^11^ ^,^ ^26^ Our case 14 presented with scrofuloderma and papulonecrotic tuberculid (Fig. 1) that promptly improved under anti-tuberculous therapy. There have been previous reports of this occurrence in patients with HIV who had varying CD4 counts and viral loads.^11^ ^,^ ^26^ ^,^ ^27^ Farrell et al. suggested that the presence of tuberculids associated with HIV infection may be related to immune dysregulation with paradoxical activation of immune responses.^27^

It is noteworthy that the patients who had a longer CTB evolution period (12 months) were previously being followed in the cohort of HIV patients and presented with chronic skin lesions that were not considered in TB diagnosis. This highlights the importance of considering CTB, to rapidly establish the correct diagnosis.

Scrofuloderma and gumma, as presented here, are considered as multibacillary forms of CTB, in which the bacillus is facilitated; these forms of CTB are associated with hosts that have immune response more deficient to the bacillus. In eight of our cases, CTB presented not only as extrapulmonary disease but also as disseminated TB. This emphasizes the need to always check for the presence of pulmonary disease, even among patients without respiratory symptoms or other suspected foci.

In this study, other forms of CTB were not observed, which is probably due to the small number of patients. However, this sample size represents the number of patients observed over 17 years at a referral centre in a city with a high TB burden. We did not observe miliary CTB, which is the second most frequently reported form in patients with advanced AIDS; we also had no cases of lichen scrofulosorum, warty TB, and lupus vulgaris, which are also reported.^10^ ^,^ ^13^ ^,^ ^15^ ^,^ ^17^

*M. tuberculosis* is the most important pathogen associated with IRIS.^28^ ^,^ ^29^ In this study, unmasking IRIS was observed in three patients with significant inflammatory signs. Due to worsening of the tuberculous lymphadenitis condition, one patient (case 15) required the use of steroids to suppress the enhanced immune response until the end of treatment. Two patients had low CD4 cell counts (< 100 cells/µL) and high baseline viral load, which are known risk factors for the development of IRIS. In a review of reported cases of TB associated with IRIS, in addition to lymphadenitis as a manifestation of IRIS, there were also reports of skin manifestations including the appearance of subcutaneous abscesses and worsening skin lesions (2.8%), as seen in our cases.^30^

Most patients (66.7%) in this study responded to the standardised regimen of 6 months of anti-tuberculous therapy. Although most (66.7%) of our patients had drug adverse reactions, all concluded the initial 6 months of therapy; the remaining patients (33.3%) required prolongation of treatment until their lesions were healed. Possible factors for those who required longer treatment time were a transitory need to interrupt the regimen owing to the side effects of medication, or the presence of IRIS and more severe forms, such as disseminated TB, which require a longer time to control the disease.

Limitations of the study were that these patients belonged to a single centre. In addition, bias may be present in the sociodemographic characteristics of patients, as well as in the collection of information using secondary data.

In this study, CTB associated with HIV infection presented as scrofuloderma of the peripheral lymph nodes or spine and solitary tuberculous gumma. Scrofuloderma of peripheral lymph nodes is diagnosed quickly due to its evolutionary characteristics, such as high visibility and symptoms. However, among patients with HIV, other subacute and chronic skin lesions are underdiagnosed and are often not valued within TB research, as we saw here in our cases of gumma. In addition, we believe that the skin provides an infertile environment for the survival and multiplication of the bacillus, as observed with the breast tissue, muscle, and spleen, even in individuals with HIV.^25^ Therefore, the diagnosis of CTB may represent a good opportunity for early identification and treatment of localised or disseminated disease.

The findings of this study can be generalised to other populations and the authors expect that these results can help to better understand and manage patients with HIV and CTB.

## References

[1] WHO - World Health Organization (2017). Global tuberculosis report.

[2] Tiberi S, Carvalho ACC, Sulis G, Vaghela D, Rendon A, Mello FCQ (2017). The cursed duet today tuberculosis and HIV-coinfection. Presse Med.

[3] MS - Ministério da Saúde (2016). Panorama da tuberculose no Brasil: a mortalidade em números. (Internet).

[4] Santos JBD, Figueiredo AR, Ferraz CE, de Oliveira MH, da Silva PG, de Medeiros VLS (2014). Cutaneous tuberculosis epidemiologic, etiopathogenic and clinical aspects - part I. An Bras Dermatol.

[5] Tappeiner G, Wolff K, Katz SI, Goldsmith LA, Gilchrest BA, Paller AS, Leffel DJ (2008). Tuberculosis and infections with atypical mycobacteria. Fitzpatrick's dermatology in general medicine.

[6] Yates V, Burns T, Breathnach S, Cox N, Griffiths C (2010). Mycobacterial infections. Rook's textbook of dermatology.

[7] Frankel A, Penrose C, Emer J (2009). Cutaneous tuberculosis a practical case report and review for the dermatologist. J Clin Aesthet Dermatol.

[8] Hinrichsen S, Moura L, Arraes L, Reis L, Lamprea D, Gava R (1996). Tuberculose cutânea e AIDS: relato de um caso. An Bras Dermatol.

[9] Fernandes C, Maltez F, Lourenço S, Morgado A, Proença R (2004). Papulonecrotic tuberculid in a human immunodeficiency virus type-1 patient with multidrug-resistant tuberculosis. J Eur Acad Dermatol Venereol.

[10] High WA, Evans CC, Hoang MP (2004). Cutaneous miliary tuberculosis in two patients with HIV infection. J Am Acad Dermatol.

[11] Akhras V, McCarthy G (2007). Papulonecrotic tuberculid in an HIV-positive patient. Int J STD AIDS.

[12] Terranova M, Padovese V, Fornari U, Morrone A (2008). Clinical and epidemiological study of cutaneous tuberculosis in Northern Ethiopia. Dermatology.

[13] Regnier S, Ouagari Z, Perez ZL, Veziris N, Bricaire F, Caumes E (2009). Cutaneous miliary resistant tuberculosis in a patient infected with human immunodeficiency virus case report and literature review. Clin Exp Dermatol.

[14] Varshney A, Goyal T (2011). Incidence of various clinico-morphological variants of cutaneous tuberculosis and HIV concurrence a study from the Indian subcontinent. Ann Saudi Med.

[15] de Azevedo TP, de Oliveira MLW (2016). Analysis of cutaneous tuberculosis cases reported from 2000 to 2013 at a university hospital in Rio de Janeiro. Rev Soc Bras Med Trop.

[16] McLachlan I, Visser WI, Jordaan HF (2016). Skin conditions in a South African tuberculosis hospital prevalence, description, and possible associations. Int J Dermatol.

[17] Spelta K, Diniz LM (2016). Cutaneous tuberculosis a 26-year retrospective study in an endemic area of tuberculosis, Vitória, Espírito Santo, Brazil. Rev Inst Med Trop São Paulo.

[18] Arianayagam AV, Ash S, Jones RR (1994). Lichen scrofulosorum in a patient with AIDS. Clin Exp Dermatol.

[19] MS - Ministério da Saúde (2011). Manual de recomendações para o controle da tuberculose no Brasil.

[20] MS - Ministério da Saúde (2018). Protocolo clínico e diretrizes terapêuticas para manejo da infecção pelo HIV em adultos.

[21] MS - Ministério da Saúde (2016). Secretaria de Vigilância em Saúde, Departamenteo de Vigilância em Saúde, Prevenção e Controle das Doenças Sexualmente Transmissíveis, Aids e Hepatites Virais. (Internet). Manual técnico para diagnóstico da infecção pelo HIV.

[22] R Core Team (2017). R: A language and environment for statistical computing. Version 3.3.3(Software).

[23] Fitzgerald DW, Sterling TR, Haas DW, Bennett JE, Dolin R, Blaser MJ (2015). Mycobacterium tuberculosis. Mandell, Douglas, and Bennett's principles and practice of infectious diseases.

[24] Held MFG, Hoppe S, Laubscher M, Mears S, Dix-Peek S, Zar HJ (2017). Epidemiology of musculoskeletal tuberculosis in an area with high disease prevalence. Asian Spine J.

[25] Tewari M, Shukla HS (2005). Breast tuberculosis diagnosis, clinical features & management. Indian J Med Res.

[26] Alsina M, Campo P, Toll A, Martinez E, Palou J, Herrero C (2000). Papulonecrotic tuberculide in a human immunodeficiency virus type 1-seropositive patient. Br J Dermatol.

[27] Farrell AM, Roberts NM, Walsh JC, Staughton RC (1996). A painful rash with AIDS. Lancet.

[28] Bell LCK, Breen R, Miller RF, Noursadeghi M, Lipman M (2015). Paradoxical reactions and immune reconstitution inflammatory syndrome in tuberculosis. Int J Infect Dis.

[29] Church LWP, Chopra A, Judson MA (2017). Paradoxical reactions and the immune reconstitution inflammatory syndrome. Microbiol Spectr.

[30] Leone S, Nicastri E, Giglio S, Narciso P, Ippolito G, Acone N (2010). Immune reconstitution inflammatory syndrome associated with Mycobacterium tuberculosis infection: a systematic review. Int J Infect Dis.

